# Reporting and methodologic quality of Cochrane Neonatal review group systematic reviews

**DOI:** 10.1186/1471-2431-9-38

**Published:** 2009-06-17

**Authors:** Khalid Al Faleh, Mohammed Al-Omran

**Affiliations:** 1 Department of Pediatrics, King Khalid University Hospital, College of Medicine, King Saud University, Riyadh, Saudi Arabia; 2 Department of Surgery, King Khalid University Hospital, College of Medicine, King Saud University, Riyadh, Saudi Arabia

## Abstract

**Background:**

The Cochrane Neonatal Review Group (CNRG) has achieved a lot with limited resources in producing high quality systematic reviews to assist clinicians in evidence-based decision-making. A formal assessment of published CNRG systematic reviews has not been undertaken; we sought to provide a comprehensive assessment of the quality of systematic reviews (both methodologic and reporting quality) published in CNRG.

**Methods:**

We selected a random sample of published CNRG systematic reviews. Items of the QUOROM statement were utilized to assess quality of reporting, while items and total scores of the Oxman-Guyatt Overview Quality Assessment Questionnaire (OQAQ) were used to assess methodologic quality. Two reviewers independently extracted data and assessed quality. A Student t-test was used to compare quality scores pre- and post-publication of the QUOROM statement.

**Results:**

Sixty-one systematic reviews were assessed. Overall, the included reviews had good quality with minor flaws based on OQAQ total scores (mean, 4.5 [0.9]; 95% CI, 4.27–4.77). However, room for improvement was noted in some areas, such as the title, abstract reporting, a *priori *plan for heterogeneity assessment and how to handle heterogeneity in case it exists, and assessment of publication bias. In addition, reporting of agreement among reviewers, documentation of trials flow, and discussion of possible biases were addressed in the review process. Reviews published post the QUOROM statement had a significantly higher quality scores.

**Conclusion:**

The systematic reviews published in the CNRG are generally of good quality with minor flaws. However, efforts should be made to improve the quality of reports. Readers must continue to assess the quality of published reports on an individual basis prior to implementing the recommendations.

## Background

As new clinical evidence is accumulating at a phenomenal rate, staying up-to-date with the current state of knowledge can be a challenging task for health care providers. It has been estimated that a physician would need to read 17–20 journal articles a day to keep up to date with his particular area of interest[1,2]. This is one of many reasons why systematic reviews and meta-analyses have been introduced as a solution to synthesize and summarize evidence of primary studies on a given topic. Meta-analyses of high quality, randomized, controlled trials (RCT) are considered to be the highest level of evidence in the hierarchy of evidence-based medicine for preventive and therapeutic interventions [3,4].

The Cochrane Collaboration (CC), founded in 1993, is a well-recognized international organization. It aims to help health care professionals make an informed decisions about health care by preparing, maintaining, and promoting the accessibility of systematic reviews on the effects of health care interventions [5,6]. The Cochrane Neonatal Review Group (CNRG), dedicated to improving outcomes of newborn infants, is one of the 51 collaborative groups registered with the CC, and has achieved a considerable amount with limited resources in synthesizing and providing the highest quality of evidence to neonatal health care providers [7]. Members of the CNRG prepare reviews of the results of RCT's of intervention for treatment and prevention of disease in newborn infants [8]. In preparing their reviews, reviewers follow systematic methods summarized in the Cochrane Reviewer's Handbook and summarized in a checklist developed specifically for the neonatal reviews by the editors of the group[9]. A protocol that outlines the specific scientific objectives and methods of the review must be accepted prior to the start of the review process. All protocols and reviews are published four times a year in the neonatal module of the Cochrane Library and on a National Institute of Child Health and Human Development (NICHD) website for Cochrane neonatal reviews. The number of reviews has steadily increased over the last few years with more than 200 completed reviews currently published.

Compared to systematic reviews published in paper-based journals, Cochrane reviews appear to have greater methodologic rigor and include elements that make them less prone to bias, such as description of inclusion and exclusion criteria, and formal assessment of the trial's quality [10]. In addition, the reviews are more frequently updated[10]. However, Cochrane reviews are not immune to methodologic flaws and the room for continual assessment and improvement in quality still exists[11]. Ensuring publication of systematic reviews with the highest possible quality will likely ensure that the results are less likely influenced by bias, therefore enabling clinicians to be more confident in implementing different interventions in their practice [12].

As for any research project, assessment of the quality of systematic reviews is essential to judge whether the recommendation warrants a change in practice. This can be done through assessment of both methodologic quality (how well the systematic review was conducted) and reporting quality (how well the methodology and findings are presented[11]. The most commonly used tools to assess quality of systematic reviews are the Quality of Reporting of Meta-analysis statement (QUOROM statement) and the Overview Quality Assessment Questionnaire-OQAQ[13,14].

The current study was performed to provide a comprehensive assessment of the quality of systematic reviews (both methodologic and reporting quality) published in CNRG and to assess whether the publication of the Quality of Reporting of Meta-analysis statement (QUOROM) published in 1999 is associated with an improvement of in the quality of the reviews under study.

## Methods

### Sample Selection

Through contact with the CNRG coordinator (Ms. Diane Haughton), we obtained all titles and first author names of all neonatal systematic reviews published in the Database of Systematic Reviews of the Cochrane Library (Issue 4, 2005). We then selected 61 reviews arbitrarily (approximately one-third of a total of 210 reviews) by a simple random sampling using Microsoft Excel 2003 random function (Microsoft Corporation, Redmond, WA, USA; table 1; [15].

**Table 1:** Characteristics of the included CNRG systematic reviews.

**Number of included reviews**	**61**
**Publication year**	
1997	3 (5)
1998	8 (13)
1999	6 (10)
2000	2 (3)
2001	6 (10)
2002	9 (15)
2003	11 (18)
2004	10 (16)
2005	6 (10)
Subspecialty *	
Cardiac	6 (10)
Respiratory	19 (31)
Neurology	1 (2)
Nutrition	14 (23)
Pain	1 (2)
Developmental care	2 (3)
Environmental	2(3)
Infectious	7 (11)
Other	9 (15)
Type *	
Therapy	61 (100)
Post-QUOROM *	44 (82)
No. of included studies**	3 (1,6)

* Numbers (percentages)

** Median + inter-quartile ranges

### Quality Assessment Instruments

Based on a previous extensive review of published scales and checklists available for quality assessment of systematic reviews[11], the two instruments selected were methodologic tools that were rigorously developed by Oxman and Guyatt (Overview Quality Assessment Questionnaire [OQAQ][14]; and the Quality of Reporting of Meta-analysis (QUOROM) statement, published in November 1999[13].

### Quality of Reporting of Meta-analysis (QUOROM) statement[13]

This tool was developed to assist the authors of systematic reviews in proper reporting. It consists of a checklist (18 items subdivided to cover; abstract, introduction, methods, results, and discussion) and a flow diagram (table 2). Since some of the items encompass sub-items, we evaluated each sub-item as a separate entity in this study.

**Table 2:** Scores of included CNRG systematic reviews based on elements of the QUOROM statement.

	QUOROM statement item *	Yes	No	Partially/ Unclear
1.	Title identified as meta-analysis or SR	0 (0)	61 (100)	
***Abstract***				
2.	Structured format used	61 (100)	0 (0)	
3.	Objectives stated	37 (61)	2 (3)	22 (36)
4.	Data sources reported	61 (100)	0 (0)	
5.	Review methods reported			
	Selection criteria	61 (100)	0 (0)	
	Validity assessment	4 (7)	57 (93)	
	Data abstraction	7 (11)	54 (89)	
	Study characteristics	1 (2)	60 (98)	
	Data synthesis	36 (59)	25 (41)	
6.	Results			
	Characteristics of studies	6 (11)	47 (89)	
	Quantitative findings	45 (85)	8 (15)	
	Subgroup	5 (9)	48 (91)	
7.	Conclusion	61 (100)	0 (0)	
8.	Introduction	61 (100)	0 (0)	
***Methods***				
9.	Searching			
	Search Terms	61 (100)	0 (0)	
	Sources			
	Electronic Databases	61 (100)	0 (0)	
	MEDLINE	61 (100)	0 (0)	
	EMBASE	32 (52)	29 (48)	
	CENTRAL	52 (85)	9 (15)	
	CINAHL	26 (43)	35 (57)	
	Others	33 (54)	28 (46)	
	Online Registry of Studies	9 (15)	52 (85)	
	Personal Files	8 (13)	53 (87)	
	Citations List	53 (87)	8 (13)	
	Hand Search of Journals	21 (34)	40 (66)	
	Proceedings	47 (77)	14 (23)	
	Authors Contacts	10 (16)	51 (84)	
	Experts Contact	26 (43)	35 (57)	
	Manufacturers	2 (3)	59 (97)	
	Restrictions			
	Year	2 (3)	58 (95)	1 (2)
	Publication Status	5 (8)	21 (35)	35 (57)
	Language	2 (3)	38 (62)	21 (34)
10.	Selection Criteria	61 (100)	0 (0)	
11.	Validity Assessment	48 (79)	13 (21)	
12.	Data Abstraction in Duplicate and Independent	58 (95)	3 (5)	
13.	Clinical Heterogeneity	8 (13)	53 (87)	
14.	Quantitative Data Synthesis			
	Principal Measure of Effect	52 (85)	9(15)	
	Method of Combining Data	40 (66)	21 (34)	
	Handling Missing Data	43 (70)	18 (30)	
	Statistical Heterogeneity	13 (21)	47 (77)	1 (2)
	Rationale for Subgroups	7 (11)	45 (74)	9 (15)
	Assessment of Publication Bias	0 (0)	61 (100)	
***Results***				
15.	Trial flow	0 (0)	61 (100)	
16.	Study characteristics	53 (100)	0 (0)	
17.	Quantitative data synthesis			
	Agreement on selection	3 (5)	57 (93)	1 (2)
	Agreement on validity	2 (4)	51 (94)	1 (2)
	Summary result	50 (94)	1 (2)	2 (4)
	Present data needed to calculate effect size	52 (98)	1 (2)	
18.	Discussion			
	Summarize key findings	60 (98)	1 (2)	
	Discuss internal and external validity	32 (53)	5 (8)	24 (39)
	Discuss potential biases	3 (5)	53 (87)	5 (8)
	Suggest future research	61 (100)	0 (0)	

* Numbers (percentages)

### Overview Quality Assessment Questionnaire (OQAQ)[14]

This tool was designed to evaluate adherence of review articles to scientific principles. It consists of 10 questions (table 3). The first 9 questions address the 5 methodologic aspects of systematic reviews including search strategy, study selection, validity assessment, data analysis, and inferences. Each of these questions was answered as follows: "yes,", "partially/can't tell,", or "no." Based on response to the 9 questions, the overall scientific quality of the review article (question 10) was graded on a 7-point scale according to the developer's instructions. The review was considered to have extensive flaws if it received a score of 1, major flaws if it received a score of 3, minor flaws if it received a score of 5, and minimal flaws if it received a score of 7[14]. The operating characteristics have been validated; including inter-rater reliability, face validity, and construct validity[16].

**Table 3:** Scores of included CNRG systematic reviews based on Overview Quality Assessment Questionnaire (OQAQ)

		Response to Question *		
**OQAQ Question**		Yes	Partially/Can't tell	No
**1**.	Search methods used to find evidence stated	61 (100)	0 (0)	0 (0)
**2**.	Search for evidence reasonable comprehensive	52 (85)	(0)	9 (15)
**3**.	Criteria used for deciding which studies to include reported	61 (100)	0 (0)	0 (0)
**4**.	Bias in the selection of studies avoided	16 (26)	36 (59)	9 (15)
**5**.	Criteria used for assessing validity of included studies reported	48 (79)	0 (0)	13 (21)
**6**.	Validity of included studies assessed appropriately	47 (77)	13 (21)	1 (2)
**7**.	Methods used to combine the findings of studies reported	37 (61)	9 (15)	15 (24)
**8**.	Findings of studies combined appropriately	31 (59)	17 (32)	5 (9)
**9**.	Conclusions made by authors supported by analysis	60 (98)	1 (2)	0 (0)
**10**.	Overall quality score	4.5 (0.9), (95% CI: 4.27, 4.77)**		

* Numbers (percentages)

** Mean (SD), 95% CI

### Data Extraction

Two reviewers (KA and MA) reviewed the full texts of all included reviews and extracted data independently in a database developed using Microsoft Access 2003 (Microsoft Corporation). Both reviewers have abstracted and analyzed the data independently. Disagreement among reviewers was resolved through consensus or by consulting a third expert adjudicator.

In addition to the items in the OQAQ and QUOROM statement, the review's demographic data were collected, including: 1) the last name of the 1^st ^author, 2) the subspecialty, 3) the epidemiologic affiliation of the 1^st ^author (whether he/she is qualified with a degree in epidemiology or research methodology), and 4) the number of included studies. Stratification based on relation to the QUOROM statement publication date was entertained to assess any potential effect of the QUOROM statement on overall quality of included reviews; however, we found that most reviews published prior to the QUOROM statement publication were updated at a later stage (we assessed the most recent version of the review).

### Pilot testing

To enhance the reviewer's inter-rater agreement, we evaluated 5 reviews (not included in the study sample) as a pilot testing of the database prior to starting the data abstraction process. Proper scoring of each item in the database was discussed in detail.

### Analysis

The primary analysis of our data was descriptive. The proportion of reports that met each criterion was determined and tabulated. Data on each item is presented as counts and percentages. To determine whether the publication of the QUOROM statement was associated with an improvement of quality of published CNRG reviews, we compared the mean and standard deviation of the overall scientific quality scores (question 10 in OQAQ) of each pre- and post-QUOROM groups using a two sample Student t-test with Minitab software version 14[15]. Since the number of reviews exceeded 30, and the data were normally distributed, a central limit theorem was assumed[15].

## Results

### Sample Demographics

Sixty-one CNRG systematic reviews were evaluated in our study. Table 1 summarizes the demographic data of the included reviews. All reviews in the CNRG addressed interventions of a therapeutic nature. The distribution of topics strongly favored respiratory (31%) and nutritional (23%) interventions. The majority of the reviews (82%) were published after the publication of the QUOROM statement. The median number and inter-quartile ranges of the included trials in each review were 3 (1, 6). Eight reviews (13%) included no studies; therefore, assessment of some elements in the results section of the reviews was unfeasible. This explains the discrepant total numbers that appear in table 2.

### Reporting Quality (QUOROM statement elements)

Reports of various sub-items of the QUOROM statement are presented in table 2. Reports ranged from 0–100%. Although all reviews included in the CNRG had the word "review" typed between brackets at the end of the title, none of the titles identified the study as a systematic review or a meta-analysis.

Almost all the CNRG review's abstracts were structured, stated objectives, reported data sources, specified selection criteria adequately, and ended with a conclusion that summarized the review's main findings. On the other hand, important abstract elements of the review's methods (validity assessment, data abstraction, and data synthesis details) and results (mainly characteristics of included studies) were inadequately reported.

As per reporting standards in the Cochrane collaboration, all reviews reported their search terms, databases searched, and any additional sources of data. The majority searched electronic databases, such as Medline and/or the Cochrane Central Register of Controlled Trials (CENTRAL) in the Cochrane Library, citation lists of included studies, and conference proceedings. However, contact with manufacturers (3%) and authors of included studies (16%) to limit the chance of publication bias were inadequately reported. Only a small number of reviews reported restrictions of the search to a certain year, language, or to published studies only.

The selection criteria, validity assessment, and the principle measure of effect were clearly and adequately stated in the methods section of the included reviews. However, most of the reviews were deficient in adequate reporting of clinical and statistical assessment of heterogeneity (21%), and providing a rational for their planned subgroup analysis (11%). None of the included reviews mentioned *a priori *plans of assessment of publication bias. The statistical effect model planned *a priori *was reported in most reviews; however, reviewers either chose a fixed effect model (64%) or reported neither fixed nor random models without adequate explanation of the rationale and whether their choice would be altered by the subsequent heterogeneity assessment of the included studies[17].

Although the majority of the reviews reported a two reviewer selection and data abstraction processes, only 3 (5%) reported agreement levels among the reviewers.

All reviews reported a detailed description of the included and excluded studies, presented summarized results, and the numbers needed to calculate the effect sizes. None of the reviews presented a chart describing the trials flow.

A summary of the review's key findings and a suggestion for a future research agenda were adequately reported in the discussion section (98%); however, a discussion of the internal and external validity issues and potential bias (5%) in the review process and methods requires further improvement.

### Methodologic Quality (OQAQ items)

Table 3 presents a summary of OQAQ items scores (items 1–9) of the included reviews. Items that were adequately reported include explicit mention of search methods, comprehensiveness of search strategy, reporting of selection criteria of included studies, methods and appropriateness of validity assessment criteria used, and a conclusion that is supported by the analysis presented in the review. Only 26% of included reviews explicitly addressed how selection bias (particularly publication bias) was avoided during the review process.

**Figure 1:**
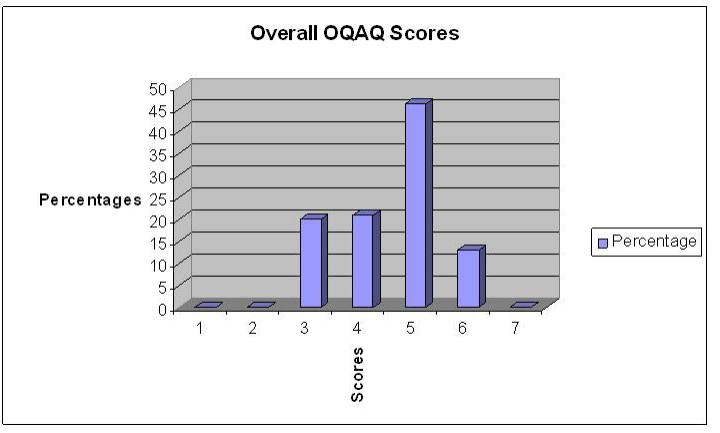
**Distribution of total OQAQ scores of CNRG systematic reviews**.

The overall quality scores (scale, 1–7; item 10) are presented in figure 1. CNRG systematic reviews scored a mean of 4.5 (0.9), (95% CI of 4.27–4.77), which translates into good quality with minor flaws of the included reviews.

### Effect of QUOROM statement publication on overall quality scores

A comparative assessment of the overall quality scores was done based on the relationship to the QUOROM statement publication (table 4). Reviews published post-QUOROM had a significantly improved total OQAQ score, with a mean difference of -1.03 (95% CI, -1.49 to -0.56, p-value < 0.0001).

**Table 4:** A comparative assessment of CNRG systematic reviews based on pre- and post-QUOROM statement status

	***Pre- QUOROM*** N = 17	***Post- QUOROM*** N = 44
Mean	3.78	4.84
Standard deviation	0.88	0.81
Min-Max	3.00 – 5.00	3.00 – 6.00
95% CI	3.34 – 4.20	4.58 – 5.08
Mean difference = -1.03 (95% CI: -1.49, -0.56), p-value < 0.0001*		

*Two sample student t-test, T-value = -4.42, DF = 59, α = 0.05

## Discussion

Our review is the first report to assess the methodologic and reporting quality of systematic reviews done in the neonatal field; our findings should be quite reassuring to neonatal practitioners. Overall, we found through the use of available instruments that reviews published in CNRG are generally of good quality; however, with minor flaws. The Cochrane reviews in general included elements that render them less prone to bias, such as a well-structured format, description of inclusion and exclusion criteria, and formal assessment of the trial's quality[10].

As mentioned above, we found that CNRG systematic reviews scored high with regards to clearly stating their objectives, comprehensiveness of search strategy, explicit detailed inclusion criteria, formal quality assessment of included trials, summarizing the key findings, and suggestion of future research topics. However, room for improvement still exists in some areas, such as the title, abstract reporting, a *priori *plan for heterogeneity assessment and how to handle heterogeneity in case it exists, assessment of publication bias, reporting of agreement among reviewers, documentation of trials flow, and discussion of possible biases in the review process.

As for most medical practitioners, health care professionals practicing in the neonatal field consider the results of systematic reviews published in the Cochrane Library of the highest level of evidence. Although the overall quality of CNRG systematic reviews was judged to be good, greater than one-third (41%) of the included reviews scored 3–4 and were considered to have major flaws, which poses an important question to the validity of the recommendations. Therefore, readers must carefully and critically appraise published reports prior to adopting a recommendation as the basis for change in practice.

We found that the quality scores of included reviews significantly improved after the publication of the QUOROM statement. This observation is prone to many biases and could simply represent improvement of research methods by time trends or improvement in the reviewer's methodological skills as they undertake more reviews.

The final quality scores reported in this review are quite comparable to the recently published review of quality of systematic review in the Cochrane Musculoskeletal Group (CMSG)[11] and higher than reported scores of systematic reviews published in anesthesia, critical care, emergency medicine, and general surgery [3,18,19], which reinforces the notion that Cochrane reviews, in general, appear to have a high methodologic quality and "frequently" updated[11].

Although we took all measures possible to enhance the validity of our results, it is important to remind the reader that our results are of an observational nature and are prone to bias. An important observation we and the authors of previous reports have noted [11,19] is the difficulty in applying the measurement tools (QUOROM statement and OQAQ), hence these instruments were not subjected to extensive validation testing hence these instruments were not subjected to extensive validation testing and lack clear and detailed guidance of their application[19]. This is particularly important in applying the overall quality score (Q10 in OQAQ), which we found to be very subjective.

## Conclusion

In conclusion, systematic reviews published in CNRG are generally of good quality with minor flaws. However, efforts should be made to improve abstract reporting, handling of heterogeneity, assessment of publication bias, and documentation of reviewer's agreement and trials flow. Readers must continue assessing the quality of published reports on an individual basis prior to implementing their recommendations.

## Competing interests

The 1^st ^author is a reviewer at the Cochrane Neonatal Review Group (CNRG).

## Authors' contributions

The authors performed the random sampling of selected reviews, developed the data collection database, conducted the statistical analysis, extracted data and evaluated the quality of included reviews independently.

## Pre-publication history

The pre-publication history for this paper can be accessed here:


